# Real world research: a complementary method to establish the effectiveness of acupuncture

**DOI:** 10.1186/s12906-015-0676-6

**Published:** 2015-05-22

**Authors:** Jing Luo, Hao Xu, Baoyan Liu

**Affiliations:** Traditional Chinese Medicine Department of Rheumatism, China-Japan Friendship Hospital, Beijing, China; Cardiovascular Diseases Centre, Xiyuan Hospital, China Academy of Chinese Medical Sciences, Xiyuan Caochang 1, Haidian District, 100091 Beijing, China; China Academy of Chinese Medical Sciences, 16 Dongzhimen South Alley, Dongcheng District, 100700 Beijing, China

**Keywords:** Acupuncture, Effectiveness, Real world research, Randomized controlled trials

## Abstract

**Background:**

Acupuncture has been widely used in the management of a variety of diseases for thousands of years, and many relevant randomized controlled trials have been published. In recent years, many randomized controlled trials have provided controversial or less-than-convincing evidence that supports the efficacy of acupuncture. The clinical effectiveness of acupuncture in Western countries remains controversial.

**Discussion:**

Acupuncture is a complex intervention involving needling components, specific non-needling components, and generic components. Common problems that have contributed to the equivocal findings in acupuncture randomized controlled trials were imperfections regarding acupuncture treatment and inappropriate placebo/sham controls. In addition, some inherent limitations were also present in the design and implementation of current acupuncture randomized controlled trials such as weak external validity. The current designs of randomized controlled trials of acupuncture need to be further developed. In contrast to examining efficacy and adverse reaction in a “sterilized” environment in a narrowly defined population, real world research assesses the effectiveness and safety of an intervention in a much wider population in real world practice. For this reason, real world research might be a feasible and meaningful method for acupuncture assessment.

**Summary:**

Randomized controlled trials are important in verifying the efficacy of acupuncture treatment, but the authors believe that real world research, if designed and conducted appropriately, can complement randomized controlled trials to establish the effectiveness of acupuncture. Furthermore, the integrative model that can incorporate randomized controlled trial and real world research which can complement each other and potentially provide more objective and persuasive evidence.

## Background

Acupuncture has been used in China over 2500 years, and is increasingly practiced in Western countries, for a variety of medical conditions, including chronic pain, hypertension, nausea and vomiting [1, 2]. The efficacy and safety profile of acupuncture has been increasingly investigated with randomized controlled trials (RCTs) over the past decades [3, 4]. Clinical research of acupuncture, and particularly the RCT, has been increasingly conducted presumably due to ongoing development of evidence based medicine. Several RCTs and relevant systematic reviews provided reasonably strong evidence that supports the efficacy of acupuncture on the treatment of some medical conditions, including pain [5], hypertension [6], seasonal allergic rhinitis [7], radiotherapy and chemotherapy induced side effects [8], etc. Other studies, including large-scale multi-center RCTs, however do not support the efficacy of acupuncture in treating pain [9–12], hypertension [13], and radiotherapy-induced emesis [14], relative to placebo or sham acupuncture. A systematic review of 13 RCTs enrolling 3025 patients evaluating acupuncture as a treatment for pain yielded inconclusive conclusion because the evidence was insufficient [15]. In general, the efficacy of acupuncture remains controversial. Table 1 is a list of examples of RCTs that did not support the efficacy of acupuncture over placebo or sham controls.

**Table 1:** Characteristics of six acupuncture randomized controlled trials with equivocal results

Trials	Conditions	Interventions	Controls	Sample size	Outcomes	Results	Practitioner
Brinkhaus B 2006 [9]	chronic low back pain	verum acupuncture	(1) minimal acupuncture; (2) waiting list	298	pain intensity (VAS), back function*, PDI, depression, QOL(SF-36), emotional aspects of pain, pain medication use	Significant difference between acupuncture and no treatment. No difference between acupuncture and minimal acupuncture.	specialized acupuncture physician
Haake M 2007 [10]	chronic low back pain	verum acupuncture	(1) sham acupuncture; (2) CT	1162	treatment response†, responder rate‡, adverse events	Significant difference between acupuncture and conventional therapy. No difference between acupuncture and sham.	experienced physician
Cherkin DC 2009 [11]	chronic low back pain	(1) individualized acupuncture; (2) standardized acupuncture	(3) simulated acupuncture; (4) CT	638	dysfunction(RDQ), symptom(bothersome score), physical and mental health(SF-36), loss of days, medication use, adverse events	Significant difference between acupuncture and conventional therapy. No difference between acupuncture and sham.	experienced acupuncturist
Vas J 2012 [12]	acute low back pain	verum acupuncture plus CT	(1) sham acupuncture plus CT; (2) placebo acupuncture plus CT; (3) CT	275	clinical improvement (RMQ), disability (RMQ), pain intensity(VAS), SEE, adverse events	Significant difference between acupuncture and no treatment. No difference between acupuncture and sham or placebo.	experienced physician
Macklin EA 2006 [13]	moderate essential hypertension	(1) individualized acupuncture; (2) standardized acupuncture	sham acupuncture	192	blood pressure, QOL, antihypertensive medication use, adverse events	No significant difference between acupuncture and sham.	licensed acupuncturist
Enblom A 2012 [14]	radiotherapy induced nausea and vomiting	verum acupuncture plus CT	sham acupuncture plus CT	215	nausea and vomiting§, SEE, adverse events	Acupuncture was effective for nausea, but no difference between acupuncture and sham.	experienced physiotherapist

Abbreviations: VAS, visual analog scale; PDI, Pain Disability Index; QOL, quality of Life; SF-36, the 36-Item Short-Form Quality of Life Questionnaire; CT, conventional treatment; RDQ, modified Roland Disability Questionnaire; RMQ, the 24-pointRoland Morris Disability Questionnaire; SEE, subjectively experienced effects

* Measured by validated German questionnaire FunktionsfragebogenHannover-Rucken

† Measured by Von Korff Chronic Pain Grade Scale or Hanover Functional Ability Questionnaire

‡ Measured by 12-item Short Form Health Survey, and patient global assessment of therapy effectiveness on a scale of 1 to 6

§ Measured by a category scale or VAS

The authors agree that evaluating acupuncture as a viable treatment modality using modern technologies, as typified by RCTs, is a major advance but believe that the current RCTs of acupuncture need to be further developed. Recently, real world research (RWR) has been conducted to estimate the effectiveness and safety of interventions in real world settings. In the current paper, we discussed the features unique to acupuncture, the existing problems that plagued clinical evaluation of acupuncture, and the feasibility of RWR for therapeutic evaluation on acupuncture. We also give some suggestions for study design on acupuncture assessment and call for high-quality RWR as a complementary method to establish the effectiveness of acupuncture.

## Discussion

### Features unique to acupuncture

**Fig. 1:**
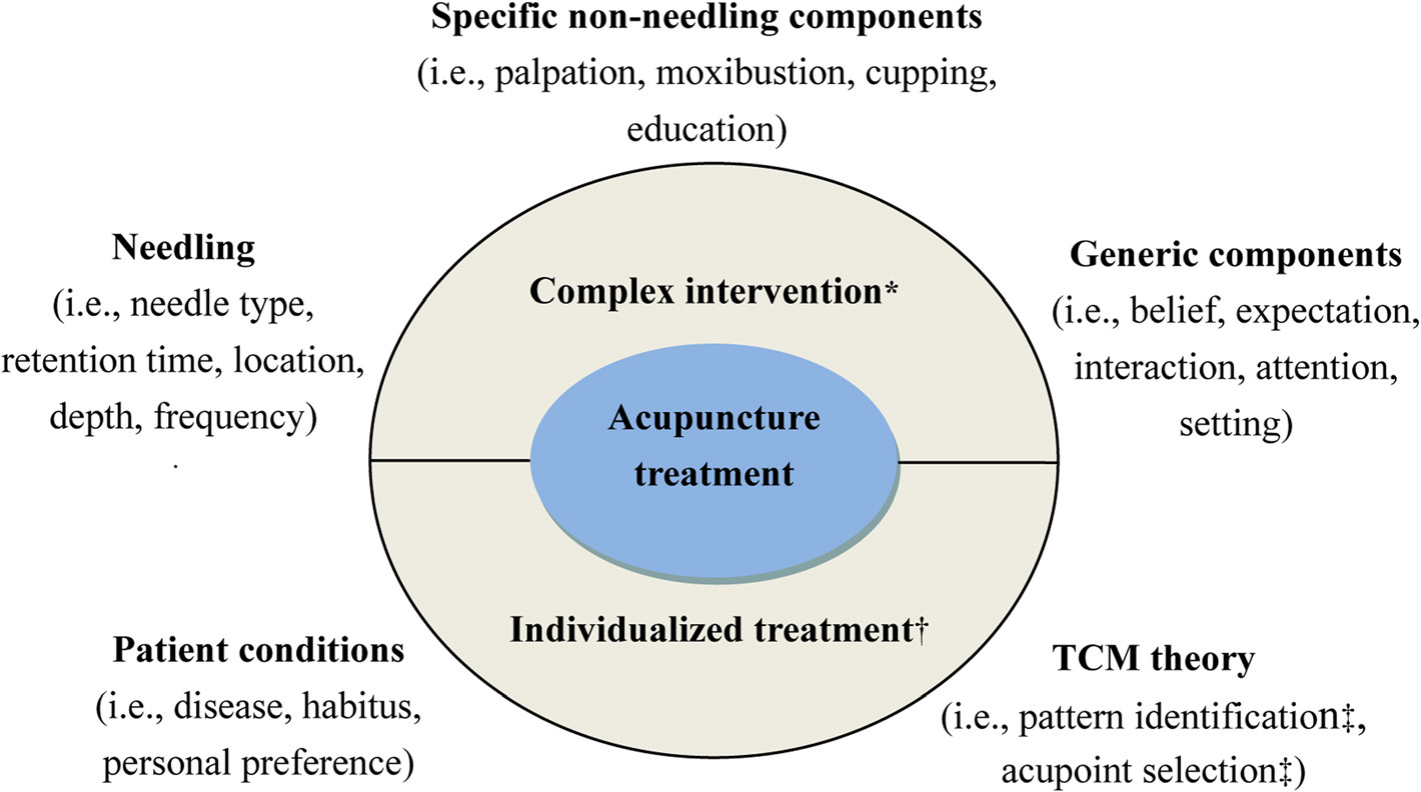
Characteristics of acupuncture treatment. Abbreviations: TCM, traditional Chinese medicine. * Acupuncture treatment is a complex intervention which includes three aspects consisting of many components. † Acupuncture treatment is an individualized treatment based on patient conditions and TCM theory. ‡ Varies considerably across different physicians as different skill and experience

Acupuncture treatment includes three aspects: needling, specific non-needling components drove by acupuncture theory, and generic components not unique to acupuncture treatment [16]. In addition, acupuncture treatment should be performed on the basis of the patient condition and traditional Chinese medicine (TCM) theory (Fig. 1).

Pattern identification refers to syndrome classification of a variety of unbalance conditions based on TCM theories. Individuals with the same disease can have distinct syndrome classification that can dictate different treatment strategies [17]. In this case, acupoint selection and regimen of treatments will be tailored to the individual patient. This situation is analogous to individualized treatment of the same condition (e.g., essential hypertension) based on the varying pathological bases in Western medicine. Generally, pattern identification and acupoint selection vary considerably across different physicians due to different skill and experience.

Within this context, instead of perceiving acupuncture as an act of merely needling, acupuncture treatment is a highly complex intervention that mainly involves pattern identification, acupoint selection, style of acupuncture, regimen of treatments, patient-practitioner interaction, and patient belief/expectation [18–20]. Moreover, acupuncture is an individualized treatment rather than a standardized needle manipulation. Clinical trials of acupuncture, therefore, should clearly define/clarify the treatment (acupuncture) characteristics (Fig. 1) to achieve objective and persuasive results.

### Benefits and limitations of RCTs on acupuncture

In evidence-based medicine, high quality RCTs and systematic reviews provide the best evidence for health care intervention [21]. In RCTs, the investigators control the factor(s) of primary interest (typically the therapeutic intervention), and therefore, could theoretically reach a reasonable conclusion with regard to cause-effect relationship. Indeed, the efficacy of acupuncture has been established with reasonable confidence in certain trials [5, 6]. However, the internal validity of RCTs is largely dependent on appropriate randomization, allocation concealment, blinding, and strict adherence to eligibility criteria. Without rigorous design and performance, results from RCTs can be unreliable. What’s more, due to the essential characteristics of acupuncture (complex intervention and individualized treatment), designing appropriate acupuncture treatments in RCTs is challenging, let alone designing and implementing appropriate placebo/sham controls for acupuncture. The following is a list of major problems in these aspects, based on the opinions of the authors as well as other scholars [22].

1. In many RCTs, acupuncture was not considered as a complex intervention that involves different components. Generally, only one or two of the whole components (typically not including pattern identification) were considered, and often in isolation [23]. Due to this imperfection, the effectiveness of verum acupuncture may be less than optimal.
2. Acupuncture in RCTs often differ considerably from that used in real world practice. A prominent problem here is lack of adjustment of the intervention based on assessment of varying pattern identification over the trial duration. This is important considering the fact that the underlying disease is a dynamic process which could be influenced by the intervention. To achieve maximum therapeutic effects, physicians often need to modify acupuncture during the trial.
3. Placebo/sham controls in acupuncture RCTs are often inappropriate. Placebo/sham controls should have similar appearance and administration, but lack the essential components of acupuncture, as stated before. However, many placebo/sham controls in acupuncture RCTs have similar characteristics such as sensory stimulation, and patient-practitioner interaction to verum acupuncture [24]. The absence of difference between acupuncture and placebos/shams could lead to underestimation of the efficacy of acupuncture. In addition, a RCT published in British Medical Journal reported higher efficacy of sham acupuncture than placebo pill on pain outcomes [25]. With this context, a paradox might emerge that verum acupuncture was superior to no treatment, but didn’t significantly outperform placebo/sham acupuncture.
4. Randomization is often difficult and sometimes not practical. Patients with a strong personal belief and preference for a specific treatment tend to refuse randomization, and are prone to dropout even after randomization.
5. Blinding is also difficult, for both investigators and patients. Experienced physicians may be able to ascertain group assignment based on their experience. Similarly, patients with prior experience to acupuncture treatment may also be able to indentify whether they receive verum acupuncture or a sham procedure.
6. Even with good internal validity, external validity (applicability to the general patient population) is often highly problematic due to unique sample characteristics and “sterilized” settings of the RCTs (relative to the real world settings) [26].

In the six RCTs listed in Table 1, the major limitations that contributed to the equivocal results supporting acupuncture actually involve imperfections regarding acupuncture treatment and inappropriate placebo/sham controls.

### Feasibility of RWR for therapeutic evaluation on acupuncture

RWR, also called observational research, is the recent name given to assess the effectiveness and safety of interventions and post-approval of pharmaceutical drugs in a real world setting [27]. In contrast to examining efficacy and adverse events in a “sterilized” environment in a narrowly defined population, RWR assesses the effectiveness and safety of a particular intervention in a much wider population in real world practice. In RWR, patients are treated with interventions in accordance with their actual conditions and preferences, rather than a random assignment. The intervention is decided by, and fully transparent to both the physicians and the patients. With regard to the study aim, RWR typically do not attempt to reach simplified/rigid treatment protocols. Another important difference (relative to RCT) is the emphasis on the patients rather than the disease; as a result, study outcomes tend to be patient-oriented or even patient-reported.

In 2002, “patient registries”, a gold standard for RWR was proposed [28]. A number of registries with large number of patients and data points, some involving international collaboration, have been developed [29–32]. For instance, the global registry of acute coronary events (GRACE) is an RWR that included 102,341 patients receiving medical care during a period between 1999 and 2009 across 154 hospitals in the world [29]. The GRACE study covered patients with acute coronary syndromes but varying co-morbidity. On the basis of clinical practice, with broad inclusion criteria, large sample size, and long term follow-up, registry studies have achieved considerable results. For example, the GRACE study in 2008 found that following presentation with a high-risk acute coronary syndrome, advanced age alone was not a contraindication to invasive management [33].

Reputable RWR on acupuncture is scarce. Due to the limitation of RCTs on acupuncture assessment, as illustrated before, we believe that RWR is much more feasible and meaningful:

1. In RWR, interventions are tailored to the patients’ specific conditions, in contrast to standardized treatment. As a result, conclusions based on RWR consider all aspects of acupuncture that affect the effectiveness.
2. At an operational level, patients’ choice of the treatment(s) decreases the difficulties in recruiting and retaining patients during the data collection period.
3. The study sample in RWR is much more representative of the real world situation (similar to the section of the population that receives the treatment). The study, therefore, has higher external validity.
4. RWR tends to have a larger sample size and longer follow-up period than RCT, and thus is more appropriate for assessing the safety of acupuncture.

By no means do we argue against RCTs for acupuncture, but we strongly believe that RWR has an important role in acupuncture research.

General limitations of RWR also apply for acupuncture RWR. First, it is not possible to firmly establish a cause-effect relationship with RWR since the intervention is not under the control of the researchers. Second, any biases and influences by confounding factors tend to be larger in RWR due to lack of control and unbalanced design. Third, effective management of a large data set is also a practical challenge.

### Suggestions for study design on acupuncture assessment

Usually upon an unexpected or unwelcome research finding, the first “culprit” tends to be poor research design and/or protocol implementation [34]. This may be particularly true for acupuncture RCTs due to the complex nature of acupuncture treatment as described above. Some efforts, e.g., the revised STandards for Reporting Interventions in Clinical Trials of Acupuncture (STRICTA) [35], have been devoted to improving reporting quality of acupuncture clinical trials. In addition, a novel statistical method (individual patient data meta-analyses) has been conducted to reanalyze data from RCTs of acupuncture and got expected and welcome findings [36]. The following is a list of additional suggestions based on the opinions of the authors as well as other scholars:

1. Acupuncture should be regarded as complex and individualized treatment [19, 37];
2. The study aim (whether to assess the efficacy of acupuncture needling or the effectiveness of acupuncture treatment) should be clearly defined and differentiated;
3. Pattern identification should be clearly specified, and non-needling components should also be considered [16, 37];
4. The treatment protocol should have some degree of flexibility to allow for individualization [38];
5. The placebo or sham acupuncture should be appropriate: knowing “what to avoid” and “what to mimic” in placebos/shams [16, 37];
6. In addition to “hard evidence”, one should consider patient-reported outcomes, economic evaluations, patient preferences and the effect of expectancy [20, 37, 39];
7. The use of qualitative research (e.g., interview) to explore some missing areas (e.g., experience of practitioners and patient-practitioner relationship) in acupuncture research [19].

**Fig. 2:**
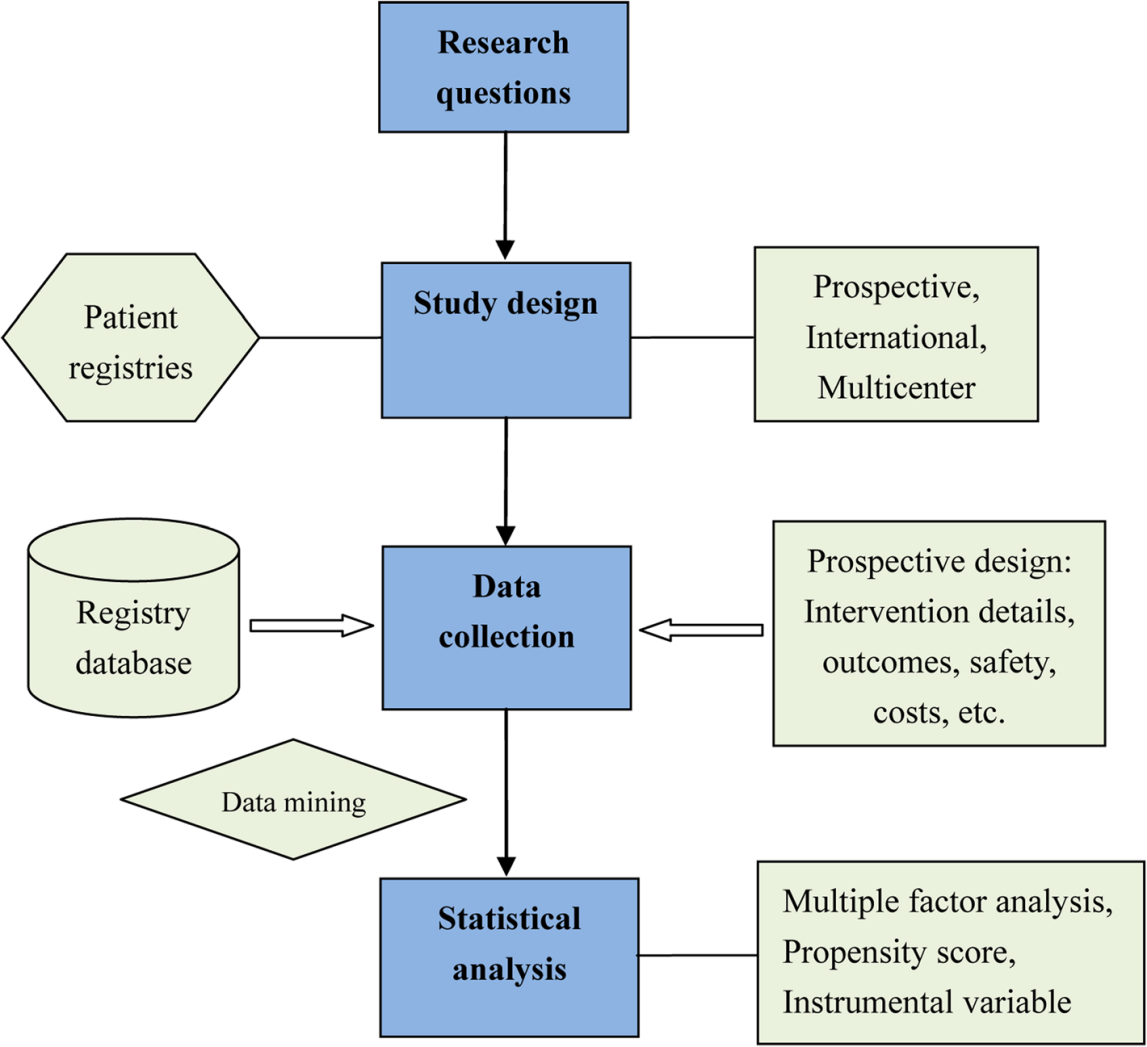
Flow diagram of real world researches on acupuncture treatment

RWR has critical importance in acupuncture study, but need to be properly designed and conducted. A suggested flow diagram of RWR on acupuncture is presented in Fig. 2.

#### Research questions

The choice of the condition(s) should be clinically significant. We believe that high-quality RWR is particularly needed for medical conditions where there are controversial evidence for acupuncture treatment, such as chronic low back pain.

#### Study design

Here, we recommend the GRACE approach [29]. Ideal RWR should be prospectively designed, and involve an international and multicenter study. In addition, information of all individual participants should be recorded in detail (including intervention, outcomes, safety and costs, as well as potential covariates) using a format that could be easily tracked.

#### Data collection

To safely guard data credibility, data collection should be prospectively designed to capture sufficient data to support analysis if possible. This is particularly important for acupuncture studies due to the complex nature of acupuncture treatment. State-of-the-art but validated technologies (e.g., data mining) for information acquisition and management of mega-database should be used [40].

#### Statistical analysis

Multiple factor analysis is almost invariably required for the complex nature of acupuncture studies. In acupuncture RWR, without rigorous inclusion criteria, randomization, and standardized treatments, potential confounding factors may often exist, and therefore, influence validity and reliability of findings. In this case, a variety of statistical techniques, such as the propensity score and instrumental variable analysis can be used to reduce/minimize the effects of confounding factors [41].

## Summary

Acupuncture is a complex and individualized treatment. Currently, the effect of acupuncture based on RCTs remains controversial. Common problems associated with acupuncture RCTs are imperfections regarding acupuncture treatment and inappropriate placebo/sham controls. RCTs are important in verifying the efficacy of acupuncture treatment, but the authors believe that RWR, if designed and conducted properly, will make a unique contribution. If taking an integrative approach, the RCT and RWR can complement each other and give full play to their advantages in acupuncture assessment. This will provide more objective and persuasive evidence in favor of acupuncture treatment.

## Abbreviations

RCTs: Randomized controlled trials
RWR: Real world research
TCM: Traditional Chinese medicine
GRACE: Global registry of acute coronary events
STRICTA: STandards for reporting interventions in clinical trials of acupuncture
